# Host Intracellular Signaling Events and Pro-inflammatory Cytokine Production in African Trypanosomiasis

**DOI:** 10.3389/fimmu.2016.00181

**Published:** 2016-05-19

**Authors:** Shiby M. Kuriakose, Rani Singh, Jude E. Uzonna

**Affiliations:** ^1^Department of Immunology, Faculty of Health Sciences, University of Manitoba, Winnipeg, MB, Canada

**Keywords:** African trypanosomiasis, intracellular signaling peptides and proteins, pro-inflammatory cytokines, innate immune response, host–parasite interactions

## Abstract

Pathogens, such as bacteria, viruses, and parasites, possess specific molecules or proteins that are recognized by several host innate immune receptors, leading to the activation of several intracellular signaling molecules and pathways. The magnitude and quality of these events significantly affect the outcome of infection. African trypanosomes, including *Trypanosoma congolense*, are capable of manipulating the host immune response, including the activity of macrophages, which are the key immune cells that contribute to the immunopathogenesis of African trypanosomiasis. Although it is known that immune hyperactivation and excessive pro-inflammatory cytokine production are the hallmarks of African trypanosomiasis, the mechanisms through which these events are triggered are poorly defined. However, it is known that macrophages may play a significant role in these processes, because phagocytosis of trypanosomes by macrophages initiates intracellular signal transduction cascades that lead to the release of pro-inflammatory cytokines and alteration in cell function. This review highlights recent progress in our understanding of the innate immune receptors, signaling pathways, and transcription factors involved in *T. congolense*-induced pro-inflammatory cytokine production in macrophages. It will reveal the existence of complex signaling events through which the parasite modulates the host immune response, thus identifying novel targets that could aid in designing strategies to effectively control the disease.

## Introduction

African trypanosomes are flagellated protozoan parasites that cause disease in both humans and animals. The disease in humans is called human African trypanosomiasis (HAT) or sleeping sickness and is considered as a neglected disease along with other parasitic diseases, such as leishmaniasis and schistosomiasis (1). Other than malaria and schistosomiasis, HAT is the third significant contributor to the global burden of parasitic diseases (1). The disease in humans is mainly caused by *Trypanosoma (T.) brucei rhodesiense* and *T. brucei gambiense*. Animal African trypanosomiasis (AAT), otherwise called Nagana, is caused by *T. congolense, T. brucei brucei*, and *T. vivax*. *T. congolense* is the major species that primarily cause animal trypanosomiasis, particularly in domestic livestock. The disease is transmitted to the mammalian host by the bite of several species of infected tsetse fly vector belonging to the genus *Glossina*. The incidence of animal trypanosomiasis coincides with the distribution of their tsetse fly vectors and according to the 2014 WHO report, tsetse fly transmitted trypanosomiasis occurs in 36 sub-Saharan countries and covers almost 10 million square kilometers of the African continent (2).

African trypanosomiasis is of considerable economic and social importance and is one of the most important factors restricting economic development in Africa (3). The disease threatens the health of about 70 million people and 50 million cattle (4). According to the 2014 WHO fact sheet, the estimated number of actual cases is 20,000, with an estimated 70 million people at risk of developing the disease (2). Due to the continued control efforts, the number of cases has dropped for the first time in last 50 years (2). However, sporadic cases and small outbreaks are reported increasingly in patients in non-endemic countries despite the decline in the number of new cases among Africans (5). It is estimated that about 50 cases of HAT occur annually outside Africa, and 94 cases of HAT were reported in 19 non-disease endemic African countries during the period of 2000–2010 (5, 6).

Although animal trypanosomiasis can occur in all domesticated animals, cattle are the main species affected due to the feeding preferences of tsetse flies. The disease adversely affects livestock production and farming and has a major impact on human and economic development. It is estimated that three million cattle die annually from the disease, and the estimated economic loss in cattle production is about 4 billion US dollars per year (7). Despite the fact that the disease kills or disables hundreds of thousands of people and animals in endemic areas, the treatment for the disease is not satisfactory. Currently, there are no vaccines available to prevent the disease in both humans and animals, and the current treatment methods have several limitations, and the development of drug resistance is a growing problem.

## Immunity to African Trypanosomiasis

### Innate Immunity

Studies have shown that various animal species respond differently to infections with various species of African trypanosomes. For example, the West African cattle breeds, such as the N’Dama, are naturally resistant (trypanotolerant) to African trypanosomiasis, whereas the zebu-type cattle breeds, such as Boran, are comparatively susceptible (trypanosusceptible). Similarly, different mouse strains show varying resistance to infections with African trypanosomes. The relatively resistant C57BL/6 represents the trypanotolerant phenotype, whereas highly susceptible Balb/c mice represent trypanosusceptible phenotype. The differential resistance and susceptibility in animals have been associated with difference in genetic factors and immune responses, including innate immunity. Both humoral (serum-associated) and cellular factors contribute to the innate immunity to African trypanosomes.

It has long been known that humans and some primates are naturally resistant to infections with certain species of African trypanosomes like *T. brucei brucei, T. congolense*, and *T. vivax* (8). This resistance is due to the presence of trypanosome lytic factors (TLF) that are present in the serum (9, 10). The key components of TLFs are apolipoprotein L-1 (APOL-1) and hepatoglobin-related protein (Hpr) (9). Evidences suggest that APOL-1 kills trypanosomes through the binding of its N-terminal domain into the parasite lysosomal membrane, leading to the generation of ionic pores (11). APOL-1 exists in two forms, the high-density lipoprotein-bound TLF, termed as TLF1, and a large lipid poor IgM/APOL-1 complex, termed as TLF2 (12, 13). These two complexes enter the parasites through different uptake modes, but their mechanism of cytotoxicity is same (14).

Apart from soluble serum factors, the innate immune cells also play a crucial role in the immunopathogenesis of African trypanosomiasis. Macrophages are perhaps one of the most important innate cells involved in this process. Studies show that the activation of macrophages in infected animals may have a dual role (protection and disease exacerbation) in African trypanosomiasis. Activated macrophages release reactive nitrogen and oxygen species that have toxic and cytostatic effects on the parasites (15, 16). In addition, macrophages also actively participate in the phagocytosis and clearance of opsonized parasites (17). However, excessive macrophage activation leads to the release of large amount of pro-inflammatory cytokines and induces immunopathology.

The activation of macrophages in African trypanosomiasis is initiated by the receptor-mediated interaction with parasite molecules that could result in phagocytosis. In particular, the interaction between the GPI membrane that anchors the parasite VSG molecule and the pattern recognition receptors (PRRs) results in macrophage activation, triggering an inflammatory response. In *T. brucei* infection, the activation of the innate immune system is mediated through the adaptor protein MyD88 (18). Although the parasite molecule that binds to macrophages in *T. congolense* infection has not been identified yet, studies from our lab have shown that immune activation is initiated by the binding of the parasite to the toll-like receptor 2 (TLR2) expressed on macrophages. This binding leads to the activation of various intracellular signaling molecules and pathways associated with inflammation. Thus, we showed that *T. congolense*-induced activation of mitogen-activated protein kinases (MAPKs) and signal transducer and activator of transcription (STAT) proteins in macrophages results in the production of various pro-inflammatory cytokines, including IL-6, IL-12, and TNF, and collectively activates the adaptive immune response. *T. congolense*-induced innate immune receptor activation and signaling events are explained later in this review.

### Adaptive Immunity

Effective immunity to any microbe requires efficient recognition of antigens derived from the microbe by the host immune system, which is followed by the generation of effector T and B cells, leading to the production of cytokines and antibodies, respectively. Due to their extracellular life style, effective immunity against African trypanosomes depends on optimal antibody production by B cells. However, this is severely hindered by the inherent ability of the parasites to undergo antigenic variation.

The variant surface glycoprotein (VSG) is a dense, protective surface coat that covers the plasma membrane of African trypanosomes. Although VSG induces strong B cell (antibody) response and anti-VSG variant-specific antibodies are effective in clearing clones expressing that specific VSG, the ability of the parasite to switch from expressing one VSG to another on its surface helps it to effectively evade the host immune system by being one step ahead of the host’s immune response (19). The constant cycling of parasites (expression of new VSGs) and antibody responses throughout the infection result in undulating parasitemia and chronic infections in African trypanosomiasis.

Since African trypanosomes are extracellular parasites, antibodies are required for protection. In *T. congolense*-infected animals, the production of antibodies against the parasite VSG occurs early after infection. Previous studies have shown that the clearance of parasites in trypanosome-infected mice is correlated with the serum levels of parasite-specific antibodies, particularly IgG2a and IgG3, but not with IgM antibodies (20). It is also clear from several studies that resistant mouse strains mount earlier and higher anti-VSG antibody response than the relatively susceptible mouse strains (20). Although anti-VSG antibodies are necessary, they are not sufficient for controlling *T. congolense* infection in the highly susceptible Balb/c mice. Interestingly, enhanced resistance to *T. congolense*-infected mice is correlated with serum levels of antibodies against common trypanosomal antigens (20).

Dysregulation of cytokine network is a hallmark of African trypanosomiasis and excessive production of inflammatory cytokines, and the release of inflammatory mediators has been proposed as a major cause of death in infected animals (20–22). The effect of cytokines in the course of trypanosomiasis is dependent on timing, microenvironment, quality, and quantity being produced. For example, type I cytokines have been shown to confer resistance to African trypanosomes by limiting parasite growth during the early stage of infection (20, 21). However, sustained type I cytokine response may be harmful and promote disease progression (23, 24). In line with this, it has been shown that anti-IFN-γ treatment resulted in very low parasitemia, control of several waves of parasitemia, and over fourfold increase in survival period in the highly susceptible BALB/c mice (25). However, infection of IFN-γ-knockout mice showed that IFN-γ is required for the survival of the relatively resistant mice to *T. congolense* or *T. brucei* infections. Thus, infected, relatively resistant C57BL/6 mice failed to control their first wave of parasitemia and succumbed acutely to the infection (21, 26). Also, the induction of IFN-γ production in CD8^+^ T cells by trypanosomal-activating molecule has been shown to lead to susceptibility (27). IL-10 is another cytokine that has been shown to be critical for trypanotolerance. Deficiency of IL-10 resulted in a striking reduction in the survival time of *T. congolense*- and *T. brucei*-infected mice, and this was associated with increased levels of serum pro-inflammatory cytokines like IFN-γ, TNF, and NO (28, 29). This has led to the conclusion that IL-10 is critically important for dampening excessive inflammatory state caused by overproduction of pro-inflammatory cytokines.

BALB/c mice are highly susceptible to experimental *T. congolense* and *T. brucei* infections, whereas C57BL/6 mice are relatively resistant. Death in the highly susceptible BALB/c mice is associated with systemic inflammatory response syndrome and shock-like state due, in part, to excessive production of pro-inflammatory cytokines and immune hyperactivation (22, 30). Thus, death of the susceptible animals is due to disease-associated complications and not primarily related to excessive parasite load itself. A recent report shows that while *T. congolense*-infected wild-type (WT) and CD4^−/−^ (CD4^+^ T cell-deficient) BALB/c mice have similar parasitemia and survival time, partial depletion of CD4^+^ T cells in WT mice leads to lower parasitemia and longer survival than infected normal WT mice (28). This partial depletion of CD4^+^ T cells also resulted in reduced IFN-γ production without affecting IL-10 and parasite-specific IgG antibody production (28). This observation further supports the notion that the early mortality of the infected BALB/c mice is due to the excessive IFN-γ production, which in turn exerts pathology by causing macrophage hyperactivation and excessive pro-inflammatory cytokine production. Thus, an optimal/balanced cytokine response is needed at the initial stage of the infection for parasite clearance, and IFN-γ-producing CD4^+^ T cells play an important role in the immunopathogenesis of African trypanosomiasis.

It has been known for long that many indigenous African mammals can harbor natural trypanosome infections without developing severe disease symptoms (31). In particular, while the indigenous West African N’Dama cattle are relatively resistant, the exotic zebu breeds are highly susceptible to African trypanosomiasis (32). The extreme susceptibility of exotic cattle breeds imported to sub-Saharan Africa may be related to excessive (rather than impaired) immune response and/or activation. Likewise, in HAT, death is usually due to the nature of the host immune reaction, as inflammatory cytokines play an important role in the development of trypanosomiasis-associated disease complications (33, 34).

Regulatory T cells (Tregs) have been shown to play an important role in the pathogenesis of several infectious diseases (35–37). However, their role in the pathogenesis of African trypanosomiasis is controversial. For example, one report suggests that Tregs play a crucial role in the enhanced resistance of mice to *T. congolense* infections (38). In this study, it was shown that CD4^+^CD25^+^Foxp3^+^ (Tregs) limit excessive IFN-γ production by CD4^+^ and CD8^+^ T cells and thereby downregulate macrophage activation and subsequent production of pro-inflammatory cytokines (38). However, overwhelming evidence from several studies suggest that Tregs may contribute to susceptibility to infection with African trypanosomes (39, 40). For example, Wei and Tabel showed that depletion of CD4^+^CD25^+^Foxp3^+^ T cells by anti-CD25 mAb leads to complete control of infection in highly susceptible BALB/c mice (39). In line with this, studies from our lab have shown that Tregs contribute to enhanced susceptibility to experimental *T. congolense* infection in mice (40). Following *T. congolense* infection, Tregs contributed to enhanced disease in both relatively resistant C57BL/6 and highly susceptible Balb/c mice. Depletion of Tregs by using anti-CD25 mAb showed that Tregs negatively affect efficient parasite control, whereas adoptive transfer of highly enriched CD4^+^ CD25^+^ Foxp3^+^ T cells resulted in increased peak parasitemia and production of disease exacerbating inflammatory cytokines like IFN-γ and IL-6 during early infection in the resistant C57BL/6 mice (40, 41).

## Role of Macrophages in African Trypanosomiasis

Macrophages play a crucial role in the control of many protozoan parasitic infections, including African trypanosomiasis. Intact monocytic cell system is important for the initiation and maintenance of anti-trypanosome responses, because the mononuclear phagocytic system is crucial in the phagocytosis of opsonized trypanosomes (13), which is the major mechanism for the removal of trypanosomes from the blood stream. During the course of trypanosome infection, the numbers of macrophages are greatly increased in many organs, including the liver, spleen, and lymph nodes, and these cells display morphological and functional features of activation (42, 43). Because phagocytosis of trypanosomes by macrophages (mainly Kupffer cells) leads to the production of pro-inflammatory cytokines (22), macrophages also play a critical role in mediating immunopathology in infected mice. The activation of macrophages during trypanosome infection is due, in part, to their exposure to parasite components and host-derived IFN-γ produced in response to parasite antigens by T cells (44). The parasite antigens include invariant membrane, cytoplasmic, nuclear antigens, and the VSG. Although the exact mechanism of attachment and phagocytosis of trypanosomes by macrophages is not fully understood, optimal phagocytosis has been shown to occur in the presence of variant-specific antibodies (45, 46).

Several studies have investigated the activation of macrophages following infection with different species of African trypanosomes. In experimental *T. brucei* infection, a large percentage of splenic macrophages exhibit membrane and functional characteristics associated with immune activation, release large amounts of IL-12, TNF, and nitric oxide, and contribute to the modulation of host immunity and resistance (47). In *T. congolense* infection, the complement and antibody-mediated phagocytosis by splenic and liver (Kupffer cells) macrophages is the major mechanism by which trypanosomes are cleared from an infected host (17, 48). The uptake of antibody-coated (including IgM and IgG) parasites by macrophages results in their activation and production of nitric oxide and pro-inflammatory cytokines (16). In addition, activated macrophages present trypanosomal antigens to CD4^+^ T cells in an MHC class II-dependent manner resulting in the production of IFN-γ (28), further enhancing macrophage activation and pro-inflammatory cytokines production. Furthermore, the massive phagocytosis of parasites by splenic and hepatic macrophages at the peak parasitemia further leads to their hyperactivation and increased production of nitric oxide, monokines, and pro-inflammatory cytokines. This systemic cytokine overproduction leads to systemic inflammatory response like syndrome, which contributes to the death of trypanosome-infected mice (28). Indeed, our group previously showed that following *T. congolense* infection, both splenic and hepatic macrophages produce copious amounts of pro-inflammatory cytokines that in turn contribute to disease and mortality (49). Treatment of infected mice with berenil (a trypanocide) significantly downregulated pro-inflammatory cytokine production and led to survival from an otherwise lethal infection in the highly susceptible BALB/c mice (49).

Direct evidence for the involvement of macrophages in the production of pro-inflammatory cytokines, following interaction with *T. congolense*, has been demonstrated *in vitro*. Kaushik et al. found that bone marrow-derived macrophages (BMDMs) produce an array of cytokines, including IL-6, TNF, and IL-12, upon stimulation with *T. congolense* and *T. brucei* whole cell lysate (50). Interestingly, they also showed that BMDMs from the highly susceptible BALB/c mice produced significantly more of these cytokines than those from the relatively resistant C57BL/6 mice (50). This is consistent with the notion that acute death observed in the infected BALB/c mice might be due to unregulated immune activation and excessive pro-inflammatory cytokine production. We have confirmed that the stimulation of BMDMs with *T. congolense* induces IL-6, IL-12, and TNF production, and this is dependent on intracellular signals resulting from activation of MAPKs and STAT proteins (STAT1 and STAT3). We further delineated the innate receptor involved in the recognition of the parasite constituent(s) and showed the critical involvement of the adaptor molecule, MyD88, in these processes.

### M1 versus M2 Macrophages

Macrophage activation is a term used to describe macrophages that have been primed or stimulated to show enhanced effector activities. Depending on the nature of activating stimuli and the eventual phenotype displayed, activated macrophages are classified into two major groups: classically activated macrophages or M1 type and alternatively activated macrophages or M2 type. The classical activation denotes effector macrophages that are produced during cell-mediated immune responses. Classically activated macrophages secreted high levels of pro-inflammatory cytokines and mediators and exhibit enhanced microbicidal and tumoricidal capacity. IFN-γ, produced by innate or adaptive immune cells, is the major molecule involved in the classical activation of macrophages (51). In contrast, Th2 cytokines, such as IL-4 and IL-13, are the key drivers of alternative macrophage activation (52). Alternatively activated macrophages are poor at antigen presentation to T cells, are less efficient at producing toxic oxygen and nitrogen radicals, and do not effectively kill intracellular pathogens (53). However, they are important in the clearance of helminth and nematode infections (54, 55).

Several studies have shown that the cytokine environment during African trypanosomiasis is key at influencing the activation of macrophages, resulting in the development of M1 and M2 subsets that are regulated antagonistically (56). Classically activated macrophages (M1) develop during the early stage of infection in the presence of type I cytokine environment (IFN-γ and TNF). In contrast, alternatively activated macrophages (M2) develop during the late stage of infection when a type II cytokine environment (IL-4, IL-10, TGF-β, etc.) predominates (56). As in other infections, M1 macrophages from mice infected with African trypanosomes have been shown to possess pro-inflammatory properties and produce nitric oxide, reactive oxygen species, and TNF, whereas M2 macrophages have anti-inflammatory properties due to increased arginase activity and IL-10 production (56). The development of M1 macrophages during the early stage of the disease appears to be responsible for the early release of pro-inflammatory mediators associated with type I immune response, and this could contribute to the control of initial parasitemic waves. Also, the activation of M1 macrophages could result in collateral damage to tissues as seen in the liver and spleen (57). A switch from M1 to M2 macrophages occurs after the clearance of initial waves of parasitemia in the relatively resistant mice (13). The resultant secretion of anti-inflammatory mediators by these M2 macrophages is thought to actively participate in tissue healing (56, 57).

## Innate Immune Receptors and Adaptor Proteins in African Trypanosomiasis

The germline-encoded innate receptor families, also known as PRRs, are critical for initiating innate immune responses in the host. They include nucleotide-binding oligomerization domain (NOD)-like receptors (NLRs), RIG-1-like receptors (RLRs), membrane-bound C-type lectin receptors (CLRs), and transmembrane toll-like receptors (TLRs). TLRs are the most widely studied PRRs and play an important role in the recognition of molecular signatures on microbes resulting in the initiation of inflammatory response (58). They are the first group of PRRs identified and have been shown to recognize a wide range of pathogen-associated molecular patterns (PAMPs) (59, 60). By inducing distinct gene expression patterns, stimulation of different TLRs leads to the activation of innate immunity, which in turn instructs the development of antigen-specific acquired immunity (61). Ten human and twelve mice functional TLRs have been identified till date, and each TLR detects distinct PAMPs derived from bacteria, viruses, fungi, and parasites (62, 63).

Toll-like receptor-mediated intracellular signaling events are initiated by the binding of different TLRs with their corresponding ligands, resulting in the recruitment of a number of adaptor proteins. Myeloid differentiation primary response protein 88 (MyD88) is the major adaptor protein that is involved in almost all TLR signaling pathways. The other adaptor proteins are TIR-domain-containing adaptor protein (TIRAP), TIR-domain-containing adaptor-inducing IFN-β (TRIF), and TRIF-related adaptor molecule (TRAM) (58, 64). The activation of these adaptor molecules, following ligation of TLRs with their specific ligands, leads to the activation of various intracellular signaling molecules (including MAPKs and STATs) and transcription factors, such as nuclear factor kappa B (NFκB) and activating protein-1 (AP-1), and a resultant initiation of specific immune responses. Mice with genetic ablation of MyD88 are highly susceptible to *T. cruzi* and *T. brucei* infections due, in part, to impaired IL-12 and IFN-γ production (18, 65). In these experimental models, it was proposed that signaling through MyD88 in innate immune cells has a protective role by activating Th1 response (18, 65). In contrast, in some experimental models, decreased pro-inflammatory responses resulting from the lack of MyD88 signaling is beneficial to the host (66). Similarly, the GPI anchor from *T. cruzi* trypomastogotes activates phosphorylation of ERK, p38, and IκB in mouse peritoneal macrophages (67), and mice deficient in the adaptor protein MyD88 signaling are highly susceptible to *T. cruzi* infection due, in part, to impaired pro-inflammatory cytokine production, suggesting that MyD88 is critical for the activation of MAPKs and NFκB by *T. cruzi* (65). Recently, we showed that deficiency of MyD88 results in inhibition of *T. congolense*-induced p38 and STAT1 phosphorylation and a concomitant downregulation of IL-6 and IL-12 by macrophages. These findings indicate that this adaptor molecule plays an important role in *T. congolense*-induced pro-inflammatory cytokine production in macrophages. They further suggest the involvement of TLR-mediated recognition and signaling in *T. congolense*-induced pro-inflammatory cytokine production in macrophages.

Although TLRs have been shown to play an important role in the innate immune responses to several parasitic infections (18, 68–71), their role in immunity to African trypanosomiasis is largely unknown. However, the findings that synergistic recognition of *T. cruzi* DNA by TLR9 and glycosylphosphatidylinositol (GPI) by TLR2 cooperatively lead to the induction of pro-inflammatory cytokine production in macrophages (72) and suggest that TLRs may play an important role in the recognition of African trypanosomes, which are related to *T. cruzi*. Indeed, a recent report showed that, in *T. brucei* infections, TLR2-mediated signaling contributes to intra-cerebral control of parasite load in the brain (73).

No report has investigated the innate receptor(s) through which the innate immune system detects and responds to *T. congolense* infection. Given that MAPKs, STATs, and NFκB pathways are activated, following stimulation of macrophages and is dependent on MyD88 signaling, we speculated that TLR(s) is/are involved in *T. congolense* recognition by macrophages. We found that the deficiency of either TLR4 or TLR9 does not affect intracellular signaling events and pro-inflammatory cytokine production in macrophages in response to *T. congolense* stimulation. In contrast, *T. congolense*-induced MAPK and STAT phosphorylation was significantly downregulated in TLR2-deficient macrophages compared to the WT cells, and this was associated with a concomitant reduction in IL-6 and IL-12 production. This indicates that *T. congolense*-induced activation of important signaling pathways leading to optimal immune response is mediated through TLR2 and may have an important role in regulating the outcome of the disease. Indeed, *T. congolense*-infected, TLR2-deficient mice on the usually relatively resistant C57BL/6 background developed uncontrolled parasitemia and died within 10 days of infection in contrast to their WT counterpart mice that controlled several waves of parasitemia and survived for more than 100 days. Altogether, these observations show that TLR2 plays a critical role in *T. congolense* recognition by macrophages and resistance to the parasite. However, it remains to be determined what parasite moiety is recognized by TLR, although the GPI, which anchors VSG to the membrane, is a prime candidate.

## Intracellular Signaling Pathways in African Trypanosomiasis

The major signaling pathways that are known to induce cytokine production in immune cells include MAPK pathway, JAK–STAT pathway, and NFκB pathway. MAPKs are a group of highly conserved serine/threonine protein kinases that mediate intracellular signaling events necessary for carrying out a variety of fundamental cellular processes, such as proliferation, differentiation, motility, stress response, apoptosis, and survival. Three major families of MAPKs are recognized including, extracellular signal-regulated kinases (ERK), p38 MAPKs, and c-Jun N-terminal kinase/stress-activated protein kinases (JNK-SAPKs) (74, 75). STATs were first described by Darnell et al. (76) as transcription factors induced by ligands in IFN-treated cells. Later, several other groups showed the critical role of STATs in signal transduction pathways by cytokines and growth factors (77). All STATs have seven well-defined domains, including an N-terminal conserved domain, dimerization domain, SH2 domain, and a C-terminal transactivation domain. The amino-terminal region prevents dimerization of STATs in their inactive state (78), and the SH2 domain is critical for the recruitment of STATs to activate receptor complexes and also for the interaction with Janus (JAK) and Src kinases. This region is the most conserved domain among STATs and plays an important role in STAT signaling by facilitating homodimerization and heterodimerization, which are crucial for nuclear localization and DNA-binding activities (79). The activation of MAPKs and STATs is important in regulating pro-inflammatory cytokine production in immune cells (80). Their activation initiates a cascade of intracellular signaling events culminating in the expression of various pro-inflammatory genes. Thus, MAPK and STAT family members coordinate and propagate multiple inflammatory immune responses (81, 82).

Nuclear factor kappa B is a family of transcription factors that play important roles in inflammation, immunity, cell proliferation, differentiation, and survival. In the inactive state, NFκB is complexed with the inhibitory protein IκBα. IκB kinase complex is activated by inducing stimuli, leading to the phosphorylation, ubiquitination, and degradation of IκB proteins. The degradation of IκB proteins releases NFκB dimers resulting in their translocation to nucleus, where they bind to specific DNA sequences and promote transcription of target genes. NFκB family is composed of five members in mammals, such as RelA/p65, RelB, c-Rel, p50, and p52. All the members are characterized by the presence of Rel homology domain (RHD), which is essential for dimerization and binding to cognate DNA elements (83).

Although infection with African trypanosomes leads to profound production of pro-inflammatory cytokines, the intracellular signaling pathways leading to the production of these cytokines are poorly studied. There are sporadic and inconsistent reports on the role of MAPK and STAT family proteins in trypanosome-induced pro-inflammatory cytokine production. Soluble VSG of *T. brucei rhodesiense* has been shown to initiate a cascade of ERK, p38, JNK MAPK, and NFκB pathways, eventually leading to the expression of various pro-inflammatory genes, such as TNF, IL-12, IL-6, and iNOS (84). Consistent with this, several protozoan infections, other than African trypanosomes, have also been shown to induce the phosphorylation of various MAPKs, leading to enhanced cytokine production. One study shows that ERK and p38 phosphorylation was triggered by *T. cruzi* (a close relative of African trypanosomes) GPI anchor, leading to the activation of NFκB and culminating in the activation of pro-inflammatory genes (67). Another report shows that the *T. cruzi* induces STAT1 phosphorylation, both at mRNA and protein levels, and this is associated with increased binding of STAT1 homodimers to gamma-activated site (GAS) elements, leading to NO production (85). In addition, another report showed that infection with *Toxoplasma gondii* leads to MAPK and STAT3 phosphorylation and subsequent production of inflammatory cytokines (86, 87). These studies suggest that MAPK and STAT family proteins may be involved in the induction of pro-inflammatory cytokine production by African trypanosomes.

Recent studies from our lab have provided strong evidence showing that MAPK and STAT proteins play a critical role in *T. congolense*-induced pro-inflammatory cytokine production in macrophages. Stimulation of immortalized macrophages and primary BMDMs with whole cell lysate of *T. congolense* leads to time-dependent phosphorylation of ERK, p38, JNK, STAT1, and STAT3 proteins, which was associated with a concomitant production of pro-inflammatory cytokines *in vivo* and *in vitro*. We confirmed the involvement of these pathways in *T. congolense*-induced pro-inflammatory cytokine production by showing that pretreatment of the cells with specific inhibitors of ERK (U0126), p38 (SB203580), JNK (SP600125), STAT1 (Fludarabine), and STAT3 (S31-201) cause a significant downregulation of IL-6 and IL-12 production in response to trypanosome stimulation^1^.

Given that the heterodimer consisting of p65 and p50 NFκB is critically important in the transcription of pro-inflammatory genes, we also assessed whether this key transcription factor is involved in *T. congolense-*induced pro-inflammatory cytokine production in macrophages. We found a time-dependent increase in the phosphorylation of NFκB p65 upon stimulation with *T. congolense* in macrophages, which corresponded with the production of IL-6 and IL-12. Collectively, our results show that the stimulation of macrophages with *T. congolense* extracts initiates intracellular signaling cascades, involving phosphorylation of MAPKs and STATs, leading to the eventual activation of the master transcription factor, of NFκB. The overall result is the transcription of pro-inflammatory genes and subsequent release of pro-inflammatory cytokines. However, it remains to be determined which innate receptor and adaptor molecules are involved in initiating these intracellular signaling events.

## Trypanosome-Induced Signaling in Relatively Resistant Versus Highly Susceptible Murine Macrophages

Although macrophages are critical for clearing parasites in the blood following infection with African trypanosomes, their classical activation and production of pro-inflammatory cytokines (IL-6, IL-12, and TNF), nitric oxide, and increased expression of MHC class II and co-stimulatory molecules (15, 44, 88, 89) have also been shown to contribute to disease pathogenesis and severity. The degree of this activation and effector function of macrophages has some relationship to the degree of susceptibility of different inbred mouse strains to experimental trypanosomiasis. Indeed, we previously reported that several cytokine genes are differentially regulated in trypanosome-infected macrophages from the highly susceptible BALB/c and relatively resistant C57BL/6 mice (50). IFN-γ-primed primary BMDMs from BALB/c mice, when infected with *T. brucei* or *T. congolense* whole cell lysate, produced significantly higher IL-6 than cells from C57BL/6 mice. Similarly, although PBMCs from both highly susceptible Boran and relatively resistant N’Dama cattle infected with *T. congolense* showed increased levels of IL-6 mRNA, this rise occurred earlier and lasted longer in the Boran than in the N’Dama cattle (90). This suggested the possibility of using IL-6 as a predictive marker of disease severity in bovine trypanosomiasis. Recent findings from our lab suggest the involvement of ERK, JAK–STAT3 pathways in *T. congolense*-induced release of IL-6 by macrophages from both BALB/c and C57BL/6 mice.

In contrast to IL-6, macrophages from BALB/c mice produced relatively lower TNF and IL-12 than those from C57BL/6 mice following trypanosome stimulation. Similar observations have been made in monocytes from the relatively resistant and highly susceptible cattle breeds (91). TNF is one of the most crucial pro-inflammatory cytokines that confer immunity against African trypanosomiasis. Magez and colleagues (92) found that the inhibition of trypanosomiasis-associated pathology in C57BL/6 and BALB/c mice is correlated with the shedding of soluble p75 TNF-receptor during peak parasitemia stages. Indeed, infection-associated pathologies were strongly reduced in both *T. brucei brucei*-infected TNF^−/−^ and TNF-R2^−/−^ mice (92). Because TNF signals through both TNF-R1 (55 kDa) and TNF-R2 (75 kDa), it has been suggested that TNF-R2 signaling in trypanosomiasis mediates infection-associated pathology, whereas TNF-R1 signaling has little or no impact on the infection outcome (92).

In addition to the production of pro-inflammatory cytokines, macrophages also produce nitric oxide, which plays a critical role in resistance against African trypanosomes *via* its cytostatic and cytotoxic effects (93–95). This is not surprising, given that the intracellular signaling pathways that result in the production of pro-inflammatory cytokines also lead to the activation of the iNOS gene and subsequent production of nitric oxide (96). Recently, we showed that in an IFN-γ-enriched environment, *T. congolense*-treated immortalized macrophages and BMDMs from the relatively resistant and highly susceptible mice show differential phosphorylation of MAPKs and STATs, suggesting that African trypanosomes induce differential signaling events in macrophages from different strains of mice *in vitro* (96). In addition to differential MAPK and STAT phosphorylation, *T. congolense* induces significantly higher levels of NO production in IFN-γ-primed immortalized macrophages and BMDMs the relatively resistant than from highly susceptible mice (96). Interestingly, we found that nitric oxide production in macrophages occurred *via* the activation of MAPK (including p38, Erk1/2, and JNK), and this was significantly inhibited by specific MAPK inhibitors in BALB/c but not in C57BL/6 cells (96). In addition, *T. congolense* and IFN-γ-induced NO production was dependent on STAT1 phosphorylation and was totally suppressed by fludarabine (a specific STAT1 inhibitor). We further showed that *T. congolense*-induced differential iNOS transcriptional promoter activation in IFN-γ-primed cells is dependent on the activation of both GAS1 and GAS2 transcription factors in BALB/c but only on GAS1 in C57BL/6 cells (96). Collectively, these observations suggest coordinated, but differentially regulated, signaling pathways that lead to NO production in macrophages from the susceptible and relatively resistant mice following their interaction with *T. congolense*.

## Concluding Remarks

**Figure 1:**
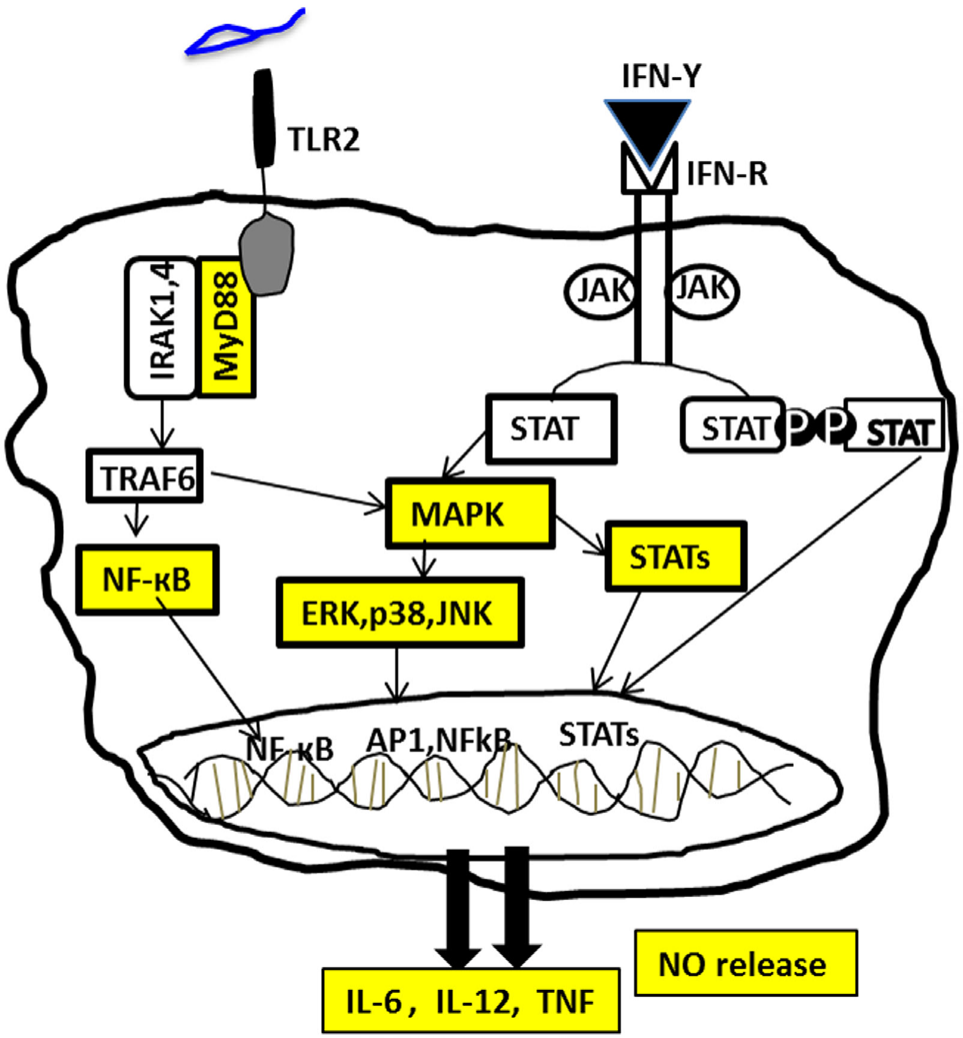
**Signaling pathways involved in *Trypanosoma congolense*-induced pro-inflammatory cytokine production in macrophages**. The recognition of *T. congolense* by macrophages through toll-like receptor 2 (TLR2) results in the recruitment of the adaptor protein MyD88 and subsequent activation of several intracellular signaling molecules, including MAPKs and STATs. Activation of these molecules in turn leads to the activation of the transcription factor NFkB, which results in the production of IL-6, IL-12, TNF, and nitric oxide (NO). Stimulation of macrophages with *T. congolense* in the presence of IFN-γ produced by CD4^+^ T cells and other immune cells further enhances the activation of these pathways, leading to increased proinflammatory cytokine and nitric oxide production.

It is clear from the experimental animal studies that susceptibility to African trypanosomiasis is related to immune hyperactivation, leading to the production of pathogenic pro-inflammatory cytokines. However, little is known about the intracellular signaling events and pathways involved in the production of these pro-inflammatory cytokines. Understanding these events could reveal novel and unexpected aspects of the parasite pathobiology that could provide new concepts for understanding host–parasite interactions, disease pathogenesis, and the major cause of susceptibility to the disease. Our recent studies clearly show the involvement of TLR2–MyD88-dependent pathway, leading to activation of MAPKs, STATs, and NFκB in *T. congolense*-induced pro-inflammatory cytokine production and resistance to the infection (Figure 1). The involvement of MAPKs and STATs in *T. congolense*-induced cytokine production suggests that targeting these pathways offer novel strategies for managing the disease in animals infected with African trypanosomes. Indeed, we have found that the beneficial effects of berenil (a trypanocide), may in addition to its trypanocidal effects, involve its ability to downregulate pro-inflammatory cytokine production *via* inhibiting intracellular signaling events, including p38, JNK MAPK, STAT1, and STAT3 phosphorylation (49, 97). Thus, targeting these pathways may be beneficial for treating disease process in animals infected with African trypanosomes. Although we have made an attempt to delineate some signaling mechanisms in macrophages, following their interaction with trypanosomal molecules, there are still important questions that remain unanswered and require extensive investigation. For example, are the host immune signaling mechanisms different in response to diverse *Trypanosoma* species and its components? How are complex signaling molecules and their interactions involved in either resistance or susceptibility of the host postinfection? What parasite antigens are recognized by TLR2, leading to activation of the innate immune system and immunity to *T. congolense* infection? Providing answers to these questions may pave the way for identification of novel targets that regulate intracellular events and pathways, leading to resistance and/or susceptibility to African trypanosomiasis.

## Author Contributions

SK and Dr. RS contributed equally to the manuscript and are co-first authors.

## Conflict of Interest Statement

The authors declare that the research was conducted in the absence of any commercial or financial relationships that could be construed as a potential conflict of interest.

## Footnotes

^1^Kuriakose et al. *Trypanosoma congolense* induced pro-inflammatory cytokine production is by the activation of TLR signaling system through MAPK and STAT proteins (submitted).
